# Human cultured IMR-32 neuronal-like and U87 glial-like cells have different patterns of toxicity under fluoride exposure

**DOI:** 10.1371/journal.pone.0251200

**Published:** 2021-06-17

**Authors:** Bruna Puty, Leonardo Oliveira Bittencourt, Iago Cesar Nogueira, Marília Afonso Rabelo Buzalaf, Edivaldo Herculano Oliveira, Rafael Rodrigues Lima

**Affiliations:** 1 Laboratory of Functional and Structural Biology, Institute of Biological Sciences, Federal University of Pará, Belém, Brazil; 2 Laboratory of Cell Culture and Cytogenetics, Environmental Section, Evandro Chagas Institute, Ananindeua, Brazil; 3 Department of Biological Sciences, Bauru Dental School, University of São Paulo, Bauru, Brazil; Xiangtan University, CHINA

## Abstract

**Background:**

Fluoride (F) is a naturally exists in nature but several studies have indicated it as an environmental toxicant to all leaving beings. Human F exposure has increased over the years since this ion has been used by industry on foods, beverages, toothpastes and on water supply. Although F is safe at optimal concentrations in water supply, human exposure to high levels could trigger neurofunctional deficits.

**Materials and methods:**

In this study, human glial-like (U87) and neuronal-like (IMR-32) cells lineages were used to access F toxicity and CNS cell sensibility on both cell facing the same protocol. Cells were exposed to F over 3, 5 and 10 days on two different F concentrations. Fluoride exposed cells were evaluated by standard toxicity assays to cell viability, apoptosis, necrosis and general cell metabolism. Oxidative stress parameters were evaluated by ATP and ROS levels, lipid peroxidation, GSH/GSSG ratio and comet assay.

**Results:**

No changes were observed in IMR-32 at any given time while after 10 days of exposure to 0.22μg/mL, U87 glial-like cells showed signs of toxicity such as decreased cell viability by necrosis while general cell metabolism was increased. Oxidative stress parameters were next evaluated only on U87 glial-like cells after 10 days of exposure. F induced a decrease on ATP levels while no changes were observed on reactive oxygen species and lipid peroxidation. GSH/GSSG ratio was decreased followed by DNA damage both on 0.22μg/mL F.

**Conclusions:**

Our results suggest an important differential behavior of the distinct types of cells exposed to the different fluoride concentrations, pointing that the U87 glial-like cells as more susceptible to damage triggered by this ion.

## Introduction

Fluoride (F) has been widely used worldwide for dental caries prevention since the 50’s. It occurs naturally in some regions on the soil and water, but can also be ingested from fluoridated public water supply, toothpastes, food, supplements and beverages [1].

After ingestion, F absorption is close to 100% in the absence of di- and trivalent cations that form insoluble compounds with F. It is well known that F absorption occurs mainly via gastrointestinal tract, with 25% being absorbed as hydrofluoric acid (HF) on stomach in an inverse relation of pH-dependent mechanism [2]. The remainder F is absorbed by the intestine, after which it crosses cellular space in its ionic form, in a no pH-dependent mechanism [2]. Once absorbed by the body, F- is carried out by the bloodstream reaching various organs and tissues [3], and 50% of the amount is stored in calcified tissues as bone and teeth. A small percentage is stored in soft tissues and some studies have suggested that F is able to cross the blood-brain barrier and accumulate in various central nervous system (CNS) regions, such as hypothalamus, cerebellum, basal ganglia, midbrain, cortex and hippo-campus [4, 5].

The most known side-effect of excessive F intake is dental/skeletal fluorosis [6]. However, some studies have linked high levels of F intake to neurological disorders, such as decreased learning and memory ability and intelligence quotient [7–11]. Moreover, studies using animal models suggest that F exposure may cause several other neurological changes, such as decreased locomotor capacity, emotional changes and decreased reflex response [8–12]. F also impairs enteric nervous system as previously reported [13, 14]. In addition, it has been shown that chronic exposure to F can cause changes in synaptic plasticity, mainly due to changes in neurotransmitters release, such as GABA, serotonin and glutamate [4, 15]. It is believed that these CNS changes may also be closely linked to increased formation of reactive oxygen species (ROS), DNA damage and cell death [16–18]. However, little is known about the effects of F effect on each type of cell of the CNS.

Composing the CNS, glial cells are mainly represented by oligodendrocytes, microglia and astrocytes, making up 90% of brain tissue in some areas [18, 19]. They are extremely important for the normal functioning of the CNS, regulating tissue homeostasis, ensuring the maintenance and survival of neurons. Additionally, these cells have the ability to release substances involved in the neuron cross-talk [19, 20]. F toxicity targets glial cells, interfering with glutamate transport and protein synthesis process [21]. Moreover, F exposure may also lead to several morphological changes on glial cells, such as cellular activation (called gliosis) and increased intracellular vacuoles [22]. While in one hand we have glial cells supporting neurons and others cerebral structures, in the other, neurons play a pivotal role in CNS features, including action potentiation, synapse transmission and neuroplasticity [23]. Along with glial cells, neurons perform memory storage and acquisition, movement commands, sensorial abilities and others [24, 25], being also subject to deleterious effects of F [26].

It is important to note that previous studies on the effect of F over the CNS models employed do not mimic human exposure, since the doses employed are far above those that can be achieved under usual chronic levels of exposure to this ion. In addition, it is important to consider that F level in plasma could be higher than the expected, if we consider that the intake of this element is influenced by a combination of different sources besides fluoridated water, such as toothpaste, food, supplements and beverages. In this way we mimic a high and long-term exposure to show whether F could be harmful to human-like CNS cells. Thus, in the present work we evaluated the effect of prolonged exposure of human glial-like cell line-age (U87 cells) and neuronal-like cell lineage (IMR-32) to F concentrations close to or higher than those found in areas of endemic fluorosis over the cell survival, metabolism, oxidative biochemistry and DNA integrity, in order to analyze the patters of response of the two different cell types to the same F dosage ranges.

## Materials and methods

### Reagents

Dulbecco’s modified Eagle’s medium (DMEM) was obtained from Invitrogen (Carlsbad, CA). Sodium Fluoride (NaF; Sigma Chemical, USA, purity >99,9%), 3-(4,5-dimethylthiazol-2-yl)-2,5-diphenyl-tetrazolium bromide (MTT), Trypan Blue, 4′,6-diamidino-2-phenylindole (DAPI) nuclear dye, TBARS assay kit and Bradford were purchased from Sigma-Aldrich. Mitochondrial Tox-Glo assay, Apotox-Glo triplex assay, ROS-Glo H_2_O_2_ assay and GSH/GSSG-Glo assay were purchased from Promega.

### Cell culture and F exposure

**Fig 1:**
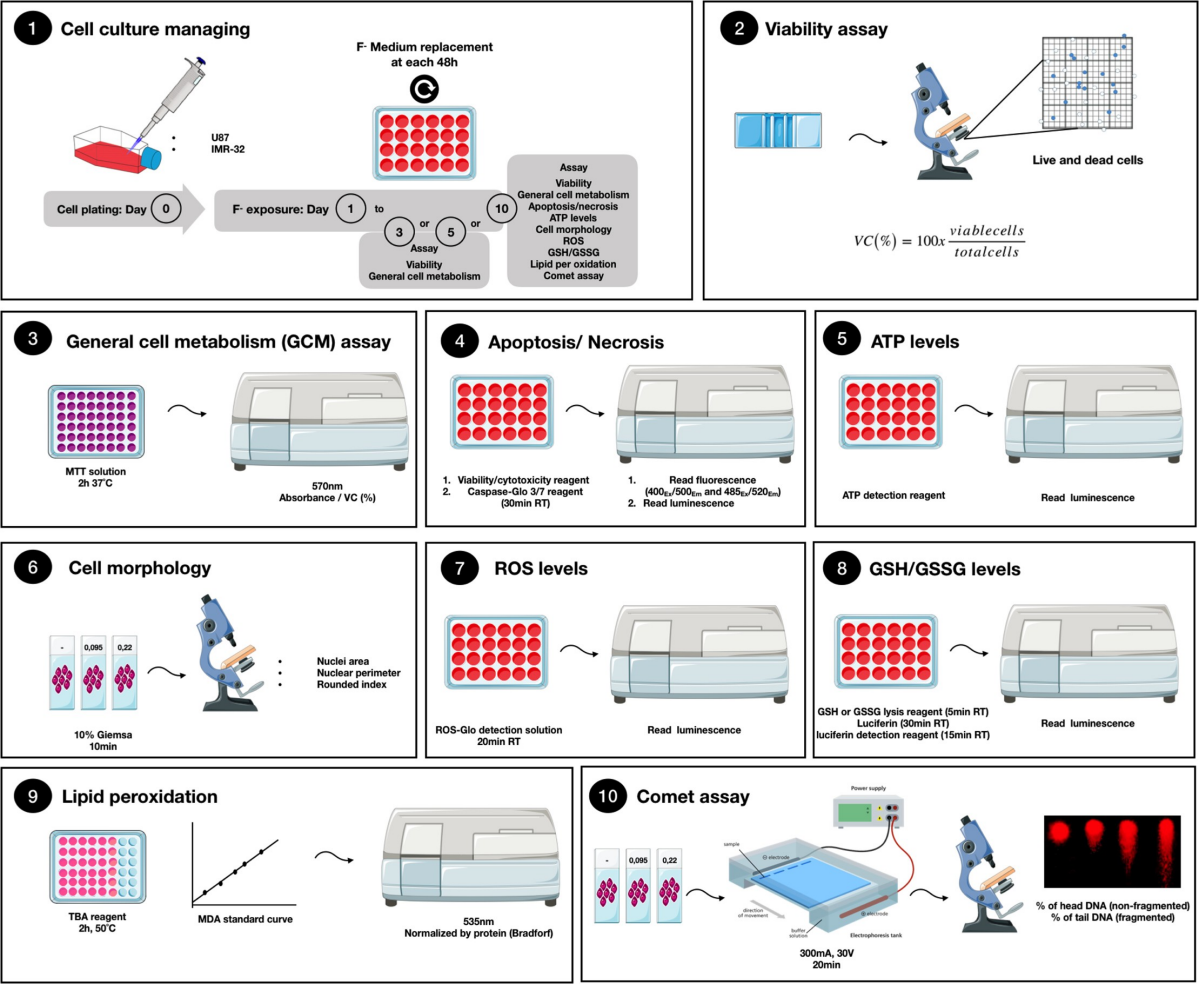
Methodological framework of cell culture and analyses. 1) U87 and IMR-32 cells were maintained in DMEM+10%FBS. Cells were exposed for 3, 5 or 10 days to a solution containing different F concentrations. After the exposure period, the following analyses were performed: 2) Viability assay; 3) General cell metabolism by MTT reduction; 4) Apoptosis/Necrosis; 5) ATP levels; 6) Cell morphology; 7) Oxidative Biochemistry assays by measuring Reactive oxygen species, 8) Oxidative Biochemistry assays by measuring GSH/GSSG ratio, 9) Oxidative Biochemistry assays by measuring Lipid Peroxidation; and 10) Comet assay.

Neuronal-like cell IMR-32 and glial-like cell U87 (ATCC) were cultured in DMEM supplemented with 10% of fetal bovine serum (FBS), penicillin (50 u/mL), streptomycin (25 μg/mL), gentamycin (25 μg/mL) and amphotericin b (2.5 μg/mL) in a controlled 5% CO_2_ and 37°C. Cells were plated onto 24-wells plate (10000 cells/well) and 96-well plates (1000 cells/well). Afterwards, cells were treated with sodium fluoride-containing medium with concentration of F equivalent to 0.095 μg/mL F and 0.22 μg/mL F, for 3, 5 or 10 days. Exposure test medium plus F was changed every two days before analyses, according to the methodological framework (Fig 1), except for cells exposed for 3 days. These F concentrations were selected to simulate plasma F levels expected to be found in people living in areas of endemic fluorosis [27, 28].

### Viability, apoptosis and necrosis assay

Cell viability was performed using trypan blue exclusion assay. After exposure, F-containing medium was withdrawn, cells washed twice with phosphate-buffered saline (PBS) and detached with Trypsin/EDTA. Cell samples were centrifuged for 3 min at 448 g. After resuspended with fresh medium, cells were counted and classified as viable and non-viable cells. The percentage of viable cells (VC) was assessed as follows:

$$VC\left(\%\right)=100x\frac{viablecells}{totalcells}$$

We also checked cell viability by Apotox-triplex assay (Promega) as well as apoptosis and necrosis according to manufacturer’s instruction. In brief, after cell exposure, 20μl of Viability/Cytotoxicity reagent containing both GF-AFC and bis-AAF-R110 substrate were added to all wells and mixed by orbital shaking (28g for 30 seconds). Samples were incubated at 37°C for 30 minutes. Fluorescence was measured using a Glomax Multi Detection System (Promega) set in two wavelengths, 400Ex/505Em (Viability) and 485Ex/520Em (Cytotoxicity—as a biomarker of necrosis). In the same wells, we added 100μl of caspase-Glo 3/7 reagent, mixed by orbital shaking (28g for 30 seconds) and incubate at room temperature for 30 minutes. Luminescence was read using a Glomax Multi Detection System (Promega).

### General cell metabolism status and ATP levels measure

The MTT assay was performed to evaluate cell metabolism. After exposure, cells were washed with PBS and MTT solution was added (100μL; 5 mg/mL) followed by incubation for 2.5 h in controlled 5% CO_2_ and 37°C. After that, MTT solution was removed and 100 μL of dimethylsulfoxide (DMSO) were added to dilute formazan crystals. Samples were read in the Glomax Multi Detection System (Promega) at 570 nm. Cell metabolism was indicated as absorbance/percentage of viable cells. Cellular ATP levels were measured by Mitochondrial Tox-Glo assay by Promega, according to manufacturer’s instruction. After exposure, assay plate was equilibrated to room temperature (10 minutes) and 100 μl of ATP detection reagent was added to each well. Samples were mixed by orbital shaking (55g) for 5 minutes. Luminescence was read in a Glomax Multi Detection System (Promega).

### Cell morphology analyses

Morphological changes were evaluated in the cultured cells after exposure. Cells were fixed with 4% paraformaldehyde (PFA) for 10min followed by 2x washing (5 min) with PBS plus glycine to inactivate PFA. Cell were washed with PBS and stained with 10% Giemsa for 10 min. The cellular morphology was observed under inverted phase contrast microscope and photomicrography were analyzed by ImageJ.

### ROS and GSH/GSSG levels

The H_2_O_2_ levels were measured by ROS-Glo H_2_O_2_ assay by Promega according to manufacturer’s instructions. In brief, before six hours after the end of F exposure, cells were treated with H_2_O_2_ substrate (25μM). After that, ROS-Glo detection solution was added to each well and samples were incubated for 20min at room temperature. Luminescence was read in a Glomax Multi Detection System (Promega). GSH/GSSG levels were measured by GSH/GSSG-Glo by Promega according to manufacturer’s instruction. The F-containing medium was withdrawal and replaced by GSH or GSSG lysis reagent. Samples were mixed by orbital shaking at 55g for 5 min. Then 50μL of luciferin were added in each well and incubated for 30 min at RT. After that, 100μL of luciferin detection reagent were added, samples were equilibrated at RT for 15min and luminescence was read in a Glomax Multi Detection System.

### Lipid peroxidation

Lipid peroxidation was assessed by following the formation of thiobarbituric acid-reactive substances (TBARS). In brief, the reagents were added in each well and incubated for 2h at 50˚C. TBAR produced a colored solution that could be read at 535nm. Sample absorbencies were compared with MDA standard curve and corrected by protein. The concentration of total protein was measured by Bradford method [29].

### Comet assay

DNA fragmentation was analyzed by the comet assay [30]. After exposure, cells were washed three times with PBS, detached with trypsin/EDTA and centrifuged for 10min at 112 g. The pellet was resuspended in 500μL of fresh medium. Twenty microliters were added to 120μL of 0.5% low-melting point agarose and transferred onto an agarose pre-coated slide. The slides were incubated at 4°C for 20 min, followed by incubation in lysis solution (1% Triton X-100, 10% DMSO, 2.5 M NaCl, 100 mM EDTA, 10 mM Tris-HCl, pH 10) for 12 h. Electrophoresis was carried out with electrophoresis buffer (300mM NaOH, 1mM EDTA, pH 13) for 20min following the setup: 300mA and 30V. After that, slides were washed three times with neutralizing solution (19.5 mM Tris-HCl, pH 7.5) for 5 min each. Cells were stained by DAPI fluorescent (15 μL; 10 μg/ml). One hundred randomly chosen cells were analyzed per sample and the plugin OpenComet at ImageJ was used to show the percentage of DNA in head (non-fragmented) or in tail (fragmented).

### Statistical analyses

All experiments were performed in triplicate (n = 3) and data were shown as median ± standard deviation. The comparisons among treatments were performed using one-way ANOVA followed by Tukey test (p<0.05). The assumptions of data normality and homogeneity of variances were previously verified by Shapiro-Wilk test.

## Results

### The fluoride induced toxicity on glial cells but not on neuronal cells

**Fig 2:**
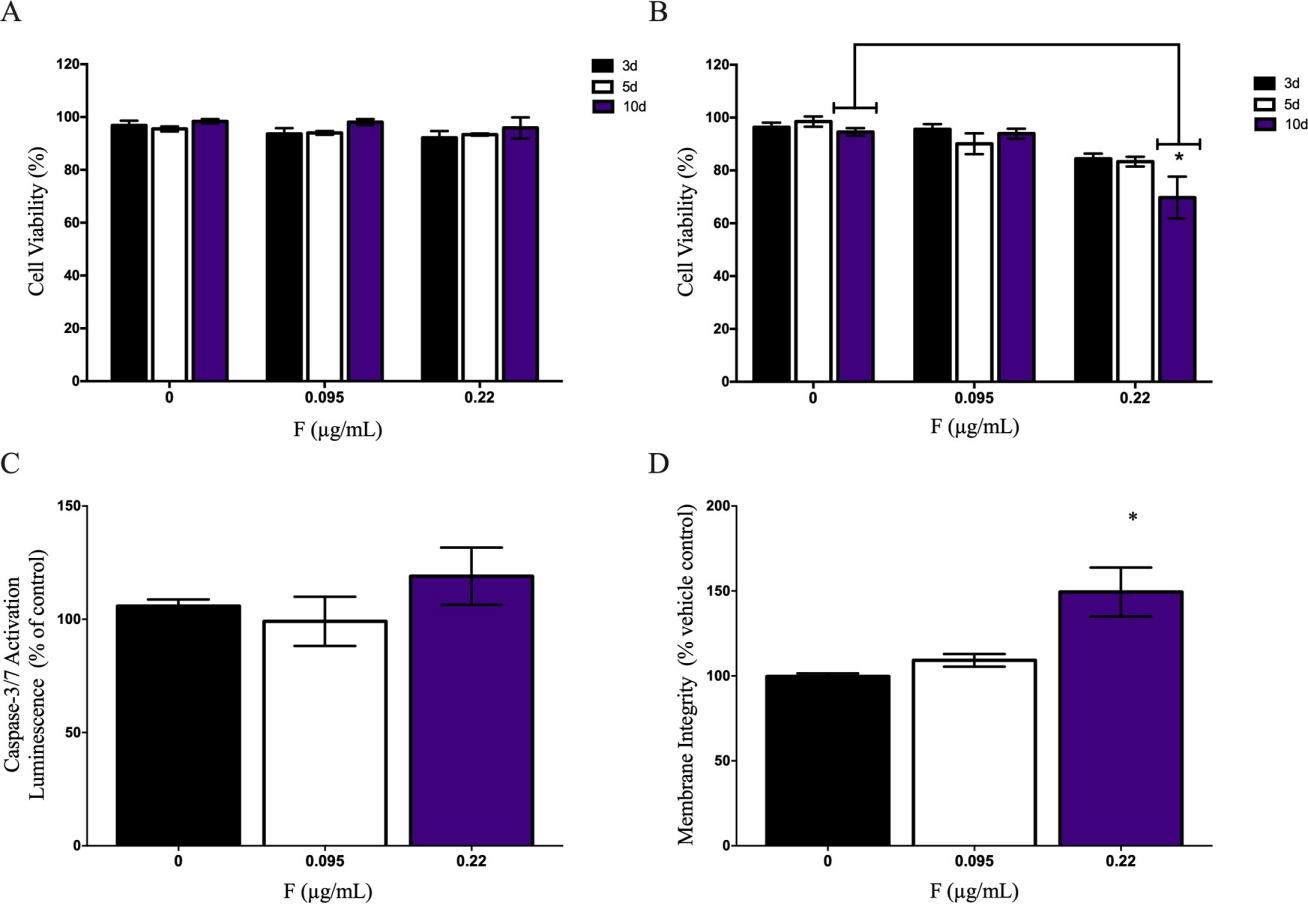
Fluoride toxicity on central nervous system human cells. Cell viability was performed on A) IMR-32 and B) U87 in different time and F concentrations. Live and dead cells were counted after 3, 5 and 10 days of F exposure. U87 cell death apoptosis or necrosis were evaluated after 10 days of 0.22μg/mL F by C) 3/7 caspase assay and D) membrane integrity analysis respectively. Data were expressed as mean ± SD. Statistical analysis were performed by one-way ANOVA followed by Tukey’s test. Statistical significance compared to control group were showed by * when p<0.05.

Neuronal (IMR-32) and glial-like cells (U87) were exposed in a temporal manner (3,5 and 10 days) to two different concentrations (0.095 μg/mL and 0.22 μg/mL) to have their sensibility to F accessed. None of the expositions caused cell death in IMR-32 cell line (Fig 2A). However, U87 cell line showed a decrease in viability when exposed to 0.22μg/mL F (66.12 ± 9.71%) after 10 days, in comparison to control (91.97 ± 5.31%) (Fig 2B). We further have analyzed induced-cell death by apoptosis or necrosis in U-87 cell line. Our results have shown a non-caspase-dependent cell death (Fig 2C), while an increased cytotoxicity as a biomarker of necrosis was observed at 0.22μg/mL (149.8 ± 25.5%) compared to control (99.71 ±3.02) (Fig 2D).

**Fig 3:**
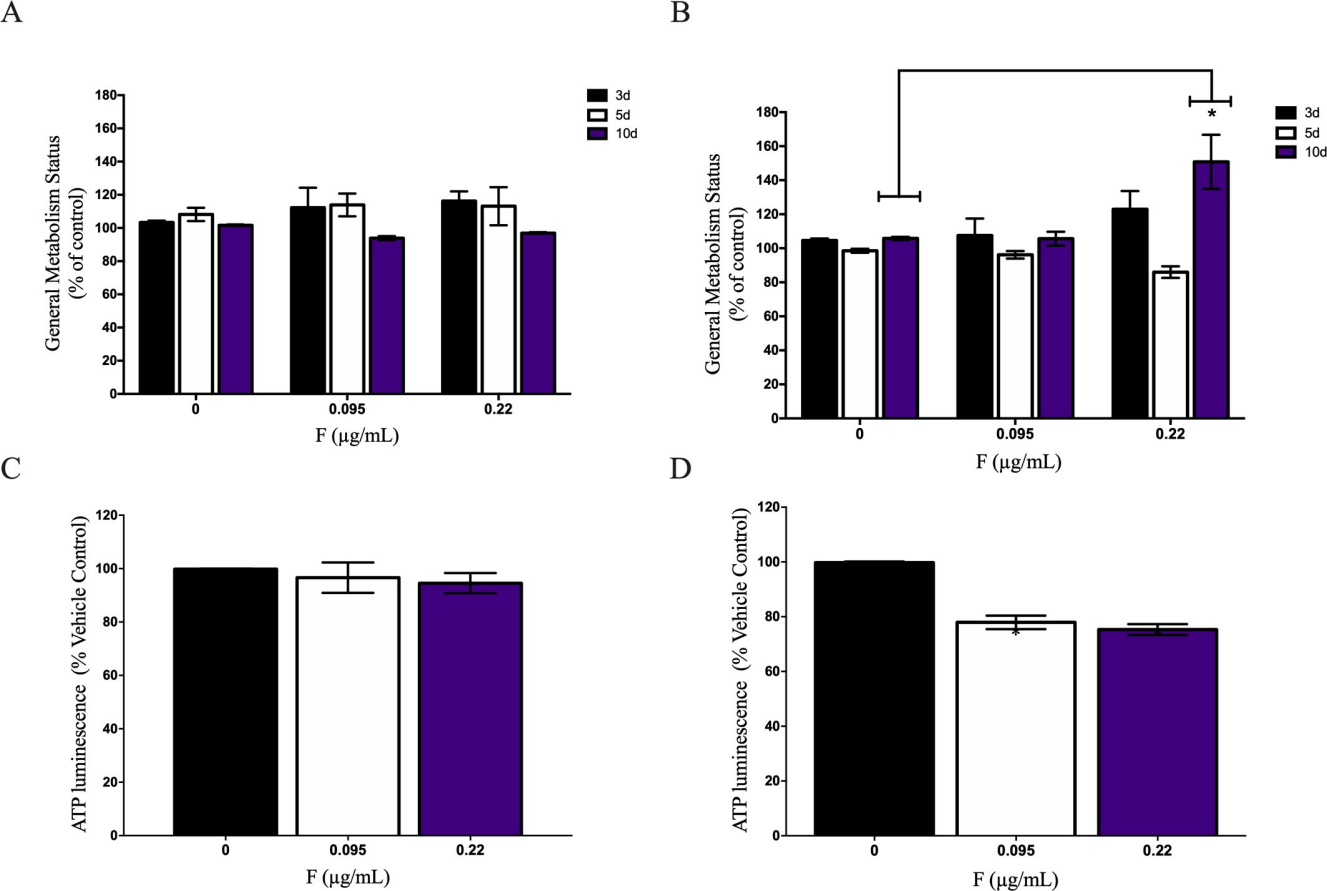
General cell metabolism (GCM) status of A) IMR-32 and B) U87 cells were performed by MTT assay in different times and F concentrations. GCM was indicated as MTT absorbance divided by percentage of viable cells. ATP synthesis was evaluated on C) IMR-32 and D) U87 after 10 days of 0.095 and 0.22μg/mL F. Data were expressed as mean ± SD. Statistical analysis was performed by one-way ANOVA followed by Tukey’s test. Statistical significance compared to control group was showed by * when p<0.05.

In the same way, general cells metabolism status was found increased only in U87 glial cells exposed for 10 days to the highest F concentration (0.22 μg/mL, 171.15 ± 27.5% vs control, 109.01 ± 6.63%) while no changes were observed on IMR-32 cells (Fig 3A and 3B). ATP levels were measured after 10 days of F exposure and our results showed no changes on IMR-32 cells (Fig 3C). On U87 glial cells, both 0.019μg/mL F (77.9 ± 2.47%) and 0.22 μg/mL F (75.31 ± 1.98%) decreased ATP levels when compared to control (99.76 ±0.4%) (Fig 3D).

**Fig 4:**
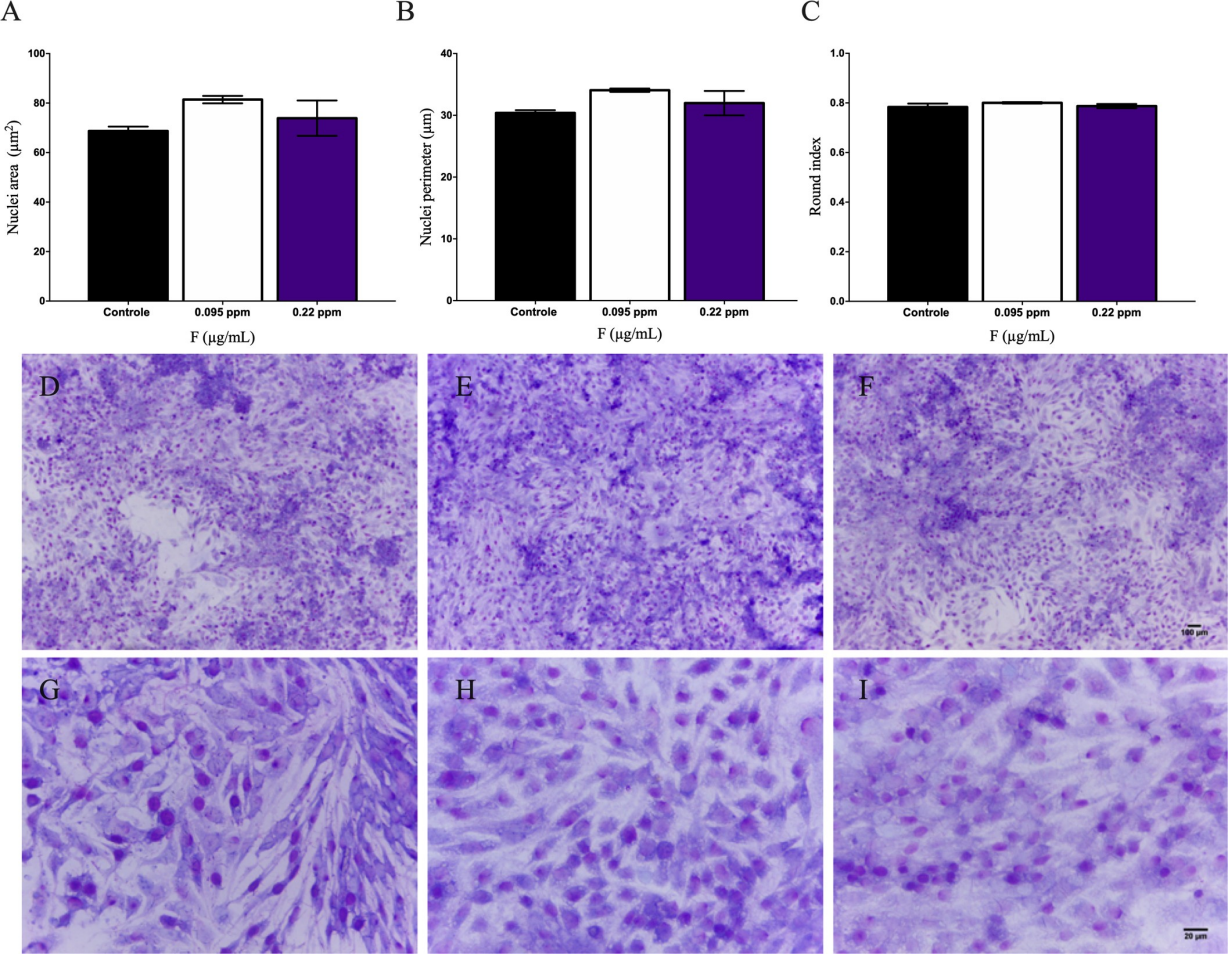
IMR-32 cells morphometric analyses after 10 days of F exposure to 0.095 and 0.22μg/mL. Cells were stained by 10% Giemsa and cellular morphology was observed under inverted phase contrast microscope and photomicrography were analyzed by ImageJ. The following parameters were evaluated: A) Nuclei area B) nuclear perimeter and C) rounded index were evaluated. Photomicrography of control (D and G), 0.095μg/mL (F and H) and 0.22μg/mL (F and I). Data were expressed as mean ± SD.

**Fig 5:**
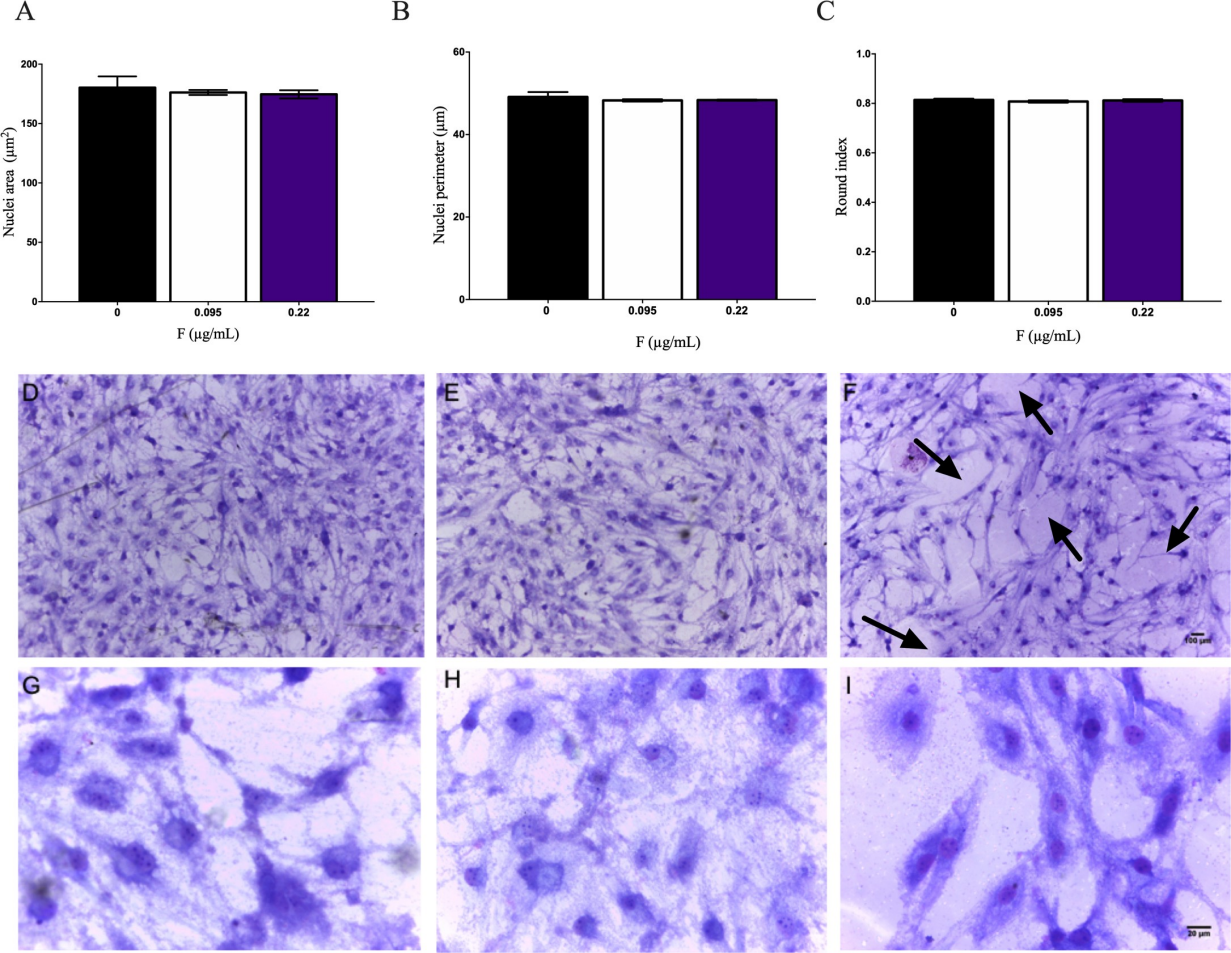
U87 cells morphometric analyses after 10 days of F exposure to 0.095 and 0.22μg/mL. Cells were stained by 10% Giemsa and cellular morphology was observed under inverted phase contrast microscope and photomicrography were analyzed by ImageJ. The following parameters were evaluated: A) Nuclei area B) nuclear perimeter and C) rounded index were evaluated. Photomicrography of control (D and G), 0.095μg/mL (F and H) and 0.22μg/mL (F and I). Data were expressed as mean ± SD. Arrows shows decreased cellular density and narrowing of cytoplasmic projections.

No changes related to nuclei area, nuclear perimeter and rounded index were observed in the morphometric analyses, in none of the cell lines (Figs 4A–4C and 5A–5C respectively). On the qualitative analysis of cell density and morphology (Figs 4D–4I and 5D–5I), a cytopathic effect was observed only in U87 glial cells in a concentration-dependent manner. Arrows show the decrease in cellular density and narrowing of cytoplasmic projections in U87 glial cells (Fig 4D–4I). As no significant changes on neuronal cells were observed, we evaluated stress parameters only in U87 glial cells after 10 days of exposure.

### Higher concentrations of fluoride reduce antioxidant parameter and cause damage to DNA integrity of glial cells

**Fig 6:**
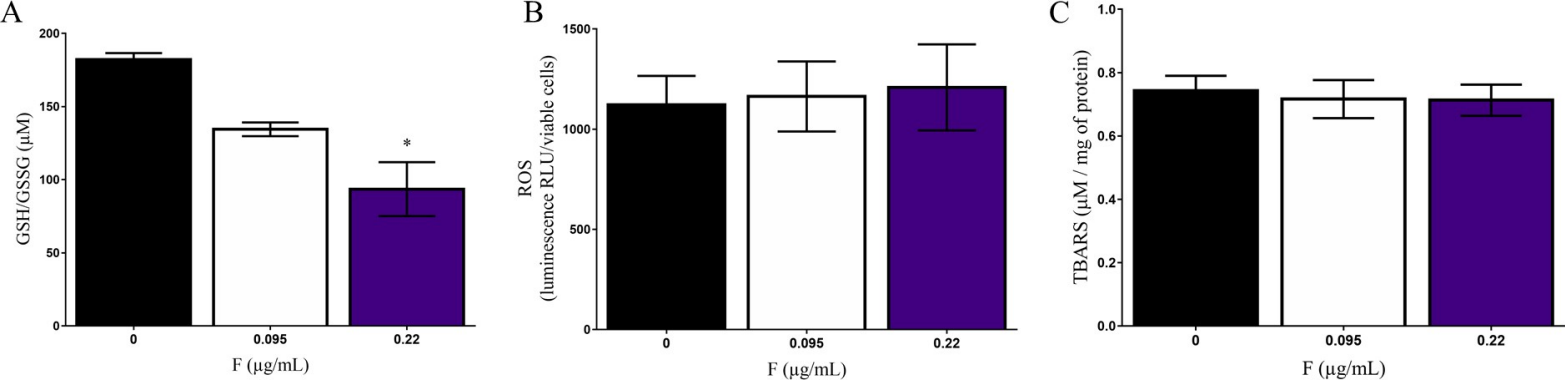
Oxidative stress parameters of fluoride exposure on U87 cells. A) GSH/GSSG ratio, B) Reactive oxygen species (ROS) and B) Lipid peroxidation (TBARS) were evaluated after 10 days of 0.095 and 0.22μg/mL F expo-sure. Data were expressed as mean ± SD. Statistical analysis were performed by one-way ANOVA followed by Tukey. Statistical significance compared to control group was showed by * whenever p<0.05.

**Fig 7:**
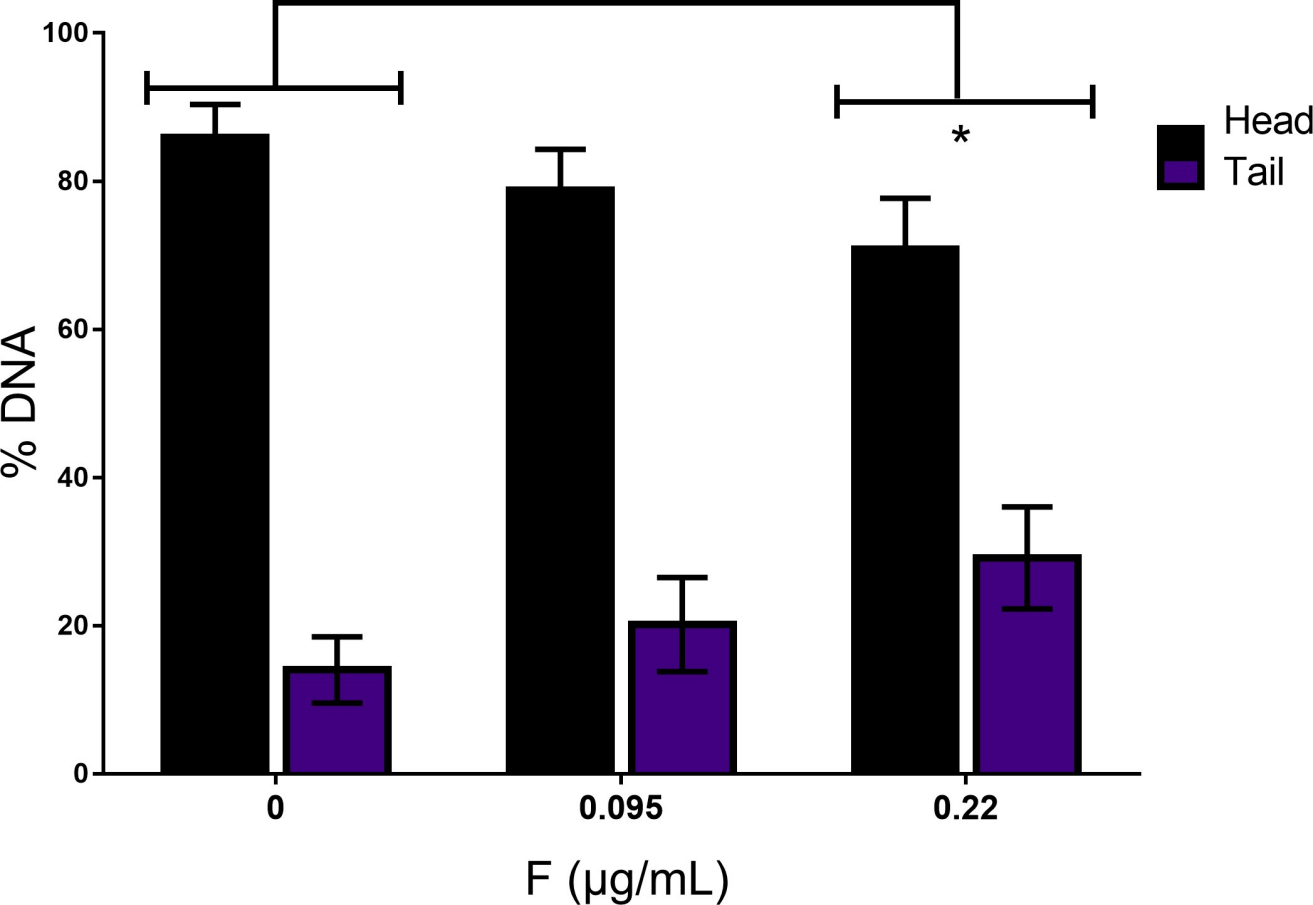
DNA fragmentation of U87 glial cells after 10 days of 0.095 and 0.22μg/mL F exposure was analyzed by comet assay. Data were showed as % of DNA on head and tail and expressed as mean ± SD. Statistical analysis were performed by one-way ANOVA followed by Tukey. Statistical significance compared to control group was showed by * whenever p<0.05.

The GSH/GSSG levels were decreased after 0.22 μg/mL (93.54 ± 16.02μM vs control, 182.19 ± 6.16μM) (Fig 6A). However, no changes in ROS levels nor in membrane lipid peroxidation were observed (Fig 6B and 6C). Our results pointed to an increase in DNA fragmentation (percentage of DNA present in the comet’s tail) when cells were exposed to 0.22 μg/mL (29.16 ± 13.99% vs control, 14.06 ± 5.3%) (Fig 7).

## Discussion

Our findings bring for the first-time evidences to the literature of the different behavior of neuronal (IMR-32) and glial-like cells (U87) facing the same range of F concentration during a short- and long-term exposures. We observed that ono major changes were observed on neuronal cells (IMR-32) exposed to F. However, under the same protocol, F exposure leads to decreased percentage of living cells, changes on spatial organization, increased general metabolism status, ATP and GSH/GSSG decreased levels as well as damage to the genetic material of U87 glial-like cells lineage when exposed to concentrations that are usually found on plasma of humans that live live in endemic areas of fluorosis, presenting high natura levels of F in the drinking water [27, 28].

To demonstrate the F-induced changes on CNS cells, we chose to use two human cell lines, a neuronal-like cell (IMR-32) and a glial-like cell (U87). Knowing that human F exposure happens constantly over long periods of life through fluoridated water, toothpaste ingestion and food consumption, we aimed to show how F leads to CNS cells toxicity over time. Thus, we first per-formed a time response curve to assess the IMR-32 and U87 glial cell F-sensitivity. Cells were exposed for 3, 5 and 10 days to solutions containing 0.095 μg/mL or 0.22 μg/mL of F and then were accessed by standard toxicity tests on cell culture, such as cell viability, general metabolism and cell morphology.

The literature about F levels in human plasma present a wide range of F concentrations, thus, several aspects must be considered, such as age, health status, methodology for F levels determination and diet. For instance, exposure of mice to 50 μg/mL of F led to plasma mean values of 0.14μg/mL [27]. When it comes to humans, Singer et al. [28] pointed that F concentrations on water supply ranging from 0.15 μg/mL to 5.4 μg/mL, led to a mean plasma concentration range from 0.14μg/mL to 0.26μg/mL, respectively. Moreover, Sener et al. [31] showed that mean plasma F levels in adult individuals consuming optimally fluoridated water was 0.017μg/mL and, as reported by Li et al. [32], individuals living in a region with high F levels in drinking water (5.03 μg/mL), presented plasma concentration of 0.106 μg/mL, while Ahmed et al. [33], showed plasma levels of 0.6μg/mL in people living in endemic regions. As mentioned above, dealing with F intake and consequent distribution requires attention to other important issues, as for example, other F sources and renal function efficiency. This is useful to illustrate that plasma concentration of F may suffers fluctuations due to several conditions, such as the intake amount and health conditions, highlighting the translational appeal of our study design, especially for those people who live in high F exposure areas.

Interestingly, no major changes were observed in IMR-32 cell at any of the exposure periods, suggesting that those concentrations used in the present work may not be harmful to this neuronal-like cell. However, when the same parameters were accessed in glial-like cells, we have observed a higher sensitivity of U87 glial-cells to F exposure, as demonstrated by the decreased cell viability and increased general cell metabolism. As no 3/7-caspase was activated, but an increased cytotoxicity was showed by loss of cellular membrane integrity, we suggest that F exposure induces U87 glial cells to primary necrosis [7, 34]. In vitro studies about F toxicity performed on CNS cells has already been demonstrated in different *in vitro* neuronal and glial/astrocyte culture models from primary origin or transformed cells line. However, it is important to note that F concentration used were generally very high and did not represent the reality of exposure of humans. Most studies have reported a cytotoxicity response approximately at 19μg/mL F a level that is far above that is found in the interstitial fluid of humans even in areas of endemic fluorosis [16, 21, 35–40].

When comparing the F-induced toxicity on neurons and glial cells, it is important to highlight that most studies have used neurons and only two have investigate de glial cells behavior after F exposure [21, 36]. The effect of F on neuronal cell viability is inconsistent across the studies with doses ranging from 18.09 μg/mL to 36.18 μg/mL while glia/astrocytes seems to shown decreased cell viability above 19 μg/mL. As our study is the first one to compare both types of cells facing the same parameters we may suggest that at those conditions presented here, U87 glial cells may respond worse to F toxicity than IMR-32 cells.

To access the F influence in cell metabolic physiology, we evaluated two parameters: the reduction of MTT by mitochondria and cytoplasmic NADPH—as an indicative of general cell metabolism status [23] and ATP synthesis [41, 42]. It is important to note that MTT method was used in this work indicate the metabolic and redox potential of U87 glial cells, and not a direct cell number or proliferation estimation (see Rai et al. [23]). Thus, after exposure to 0.22μg/mL for 10 days, U87 glial cells showed a higher general cell metabolism or redox status when compared to control group, indicating possibly a protective mechanism in order to maintain the normal cell physiology under F exposure. On the other hand, ATP synthesis was decreased after 10 days of F exposure on U87 glial cells both at 0.019 and 0.22μg/mL. Several studies have already shown that F can act as a mitochondrial toxin in both animal and cell culture models, but the full understanding of how this ion damages the mitochondrial remains unknown [40, 43–47]. Mitochondrial changes lead to impairment on normal respiratory chain function mediated by Na+/K+-ATPase inhibition and decreased ATP production [48–51]. Interestingly, our results have shown in accordance, a significant influence of F in mitochondrial mechanism observed in U87 glial cells and in vivo experiments, as a recent study reported profound alterations in the mitochondrial protein profile of rats exposed to water containing 50 μg/mL F for 15 days [52], leading to plasma F levels close to the lowest dose evaluated in the present study (0.095 μg/mL). In this way, we believe that glial cells could be a suitable model to understand the F mode of action as a mitochondrial toxin, although additional studies are necessary to clarify that. As no major primary changes were observed in IMR-32 cells, we continued the F toxicity investigation only using the U87 glial cells exposed to F for 10 days.

It is important to note that in the present study the cells were cultured alone, losing the glial-neuron interaction but this first assessment on isolated cells may lead us to understand the order of magnitude where F toxicity is expected on CNS cells. As mentioned earlier, glial cells are not only supportive nutritional cell for neurons but have an important role mediating neuronal communication at CNS with direct influence on neurotransmitter release and recycle [19, 20]. Thus, changes on glial cells normal physiology may lead to important changes on the glial-neuron crosstalk and CNS connectivity, which may be linked to CNS dysfunction over F toxicity in vivo model and in human [7–11].

Because the metabolic evaluation has suggested an increased redox potential of U87 glial cells under F toxicity and several studies have hypothesized that neurological damages after F consumption may be related to oxidative stress [52–54] we, then, sought to assess whether the toxic effects observed in U87 glial cells would be due to oxidative-induced misbalance. Oxidative stress biomarkers after 10 days of exposure were performed by measuring H_2_O_2_ levels, since it is well recognized that H_2_O_2_ is the final product of other ROS and has the longest half-life. We have also analyzed membrane lipid peroxidation, GSH/GSSG levels and DNA damage. We did not have identified changes in ROS levels and membrane lipid peroxidation. However, GSH/GSSG levels decreased after exposure to 0.22 μg/mL. As glutathione is an important antioxidant enzyme that minimizes free radicals, it is possible that F exposure leads to a slight increase in ROS that was buffered by GSH. In the study proposed by Araújo et al. [52] a similar effect of F exposure was observed on the highest dose, showing an increased energy metabolism and a reduction on antioxidant enzymes, such as Superoxide dismutase (SOD), Glutathione peroxidase (GPx) and Catalase (CAT). Thus, our results seem to be in accordance with previous data from literature suggesting that the effects of F on energy cell metabolism depends on the time of exposure and F concentration [52]. In addition, decreased GSH/GSSG could be also a result of changes in ATP levels as glutathione synthesis occurs through two enzymatic reactions that are ATP-dependent. Then, in situations in which there is a decrease in ATP production, as showed by our results, it is possible that changes in the glutathione system may occur without necessarily relation to increased ROS levels [55]. Thus, we can hypothesize that the antioxidant system of U87 glial cells by GSH/GSSG conversion reduced ROS induction by F. Another evidence of oxidative stress could be by the DNA damage of U87 glial cells after F exposure. Our results pointed to an increase in DNA fragmentation (percentage of DNA present in the comet’s tail) when the cells were exposed to 0.22 μg/mL. This fragmentation appears independently of apoptosis-mediated cell death (see Fig 2C), so it is important to note that this DNA fragmentation may function as an indicator of necrosis that occurred after days of exposure to a cytotoxic compound, corroborating the results previously shown. Recent studies have suggested that the boundary between the different characteristics of apoptosis- or necrosis-mediated death has been increasingly difficult to observe [7, 56, 57]. In a study performed on Jukart cells it was shown that in the early stages of necrosis it was possible to observe DNA fragmentation specifically by cutting only at the 5 ’ends. The membrane rupture during necrosis may be able to release nucleases responsible to cause damage to the own genetic material. However, the mechanisms underlying of this type of fragmentation are not fully understood yet [58, 59].

Campos-Pereira et al. [60] have shown a similar effect of long-term F exposure on rat hepatocytes. These authors have shown an increase on oxidative stress and DNA fragmentation that is able to disrupt cellular homeostasis. This is in accordance with results presented in the present study, suggesting that F exposure could also compromise CNS cells. It is important to highlight that only the lowest F concentration evaluated in the present study (0.095 μg/mL) is similar to levels of F observed in plasma from individuals living in endemic areas of fluorosis, based on several studies conducted with rodents that received water containing 50 mg/mL of F [13, 27, 54]. Despite some studies have reported levels of F in plasma as high as 0.6 μg/mL in areas of endemic areas, the method of analysis in these studies can be questioned [33]. Thus, the only toxic effect found in the present study that could be expected to occur in areas of endemic fluorosis (up to 10 μg/mL F in the drinking water) is reduction in ATP production, which is inline with a previous study conducted with rats drinking water containing 50 μg/mL F for 15 days [52]. However, it is known that the effects of F in the organism are not only dose- but also time-dependent [54]. Araújo et al. [52] reported profound alterations in proteins involved in energy metabolism were found in mitochondria of rats treated with water containing 50 μg/mL F for 15 days, but when the treatment was extended to 60 days, the alterations were much smoother, indicating an adaptation of the organism to the effects of F. Thus, additional studies should be conducted implying longer periods of treatment and also lower doses of F. It would be of particular interest to conduct long-term studies using F concentrations that lead to plasma F levels similar to the ones found in humans drinking optimally fluoridated water.

## Conclusions

Our data allow us a first assessment of F toxicity on two CNS cells lineage facing the same protocol to understand the concentration where adverse effects may be expected on each isolated cells. U87 glial-like cells seems to be more susceptible in comparison to IMR-32 neuron-like cells. This susceptibility is observed in a dose-dependent manner, in which lower and higher concentrations of F can impair different biological processes and cellular components, including DNA injurie. More studies are required to investigate the behavior of both cells in the same microenvironment, by coculture, for example, however our results give start for further investigations and reinforce that F as an environmental pollutant must be carefully managed.

## Supporting information

S1 Table Curve time-response on IMR-32 and U87 cells for 3,5 and 10 days of F exposure (cell viability and general cell metabolism). Further analysis to access fluoride toxicity were performed only after 10 days of F exposure (ATP, Membrane integrity and cascade 3/7 and Morphometric analysis’ (nuclei area, nuclei perimeter and round index)). Oxidative stress parameters were evaluated only on U87 glial cells after 10 days of exposure (ROS, TBARS, GSH/GSSG, DNA damage). Results are expressed as mean and SD: Standard deviation.
(DOCX)
