# Supplementary material for: Human cultured IMR-32 neuronal-like and U87 glial-like cells have different patterns of toxicity under fluoride exposure
Source: PLoS One. 2021 Jun 17;16(6):e0251200. doi: 10.1371/journal.pone.0251200 (PMC8211231; doi:10.1371/journal.pone.0251200)
Supplement: S1 Table — Further analysis to access fluoride toxicity were performed only after 10 days of F exposure (ATP, Membrane integrity and cascade 3/7 and Morphometric analysis’ (nuclei area, nuclei perimeter and round index)). Oxidative stress parameters were evaluated only on U87 glial cells after 10 days of exposure (ROS, TBARS, GSH/GSSG, DNA damage). Results are expressed as mean and SD: Standard deviation. (DOCX) [file pone.0251200.s001.docx]

| **Analyze** | **Description of unit** | **Mean (Control)** | | | **Mean (0.095µg/mL)** | | | **Mean (0.22/mL)** | | | **SD (Control)** | | | **SD (0.095µg/mL)** | | | **SD (0.22µg/mL)** | | |
| --- | --- | --- | --- | --- | --- | --- | --- | --- | --- | --- | --- | --- | --- | --- | --- | --- | --- | --- | --- |
|  |  | 3d | 5d | 10d | 3d | 5d | 10d | 3d | 5d | 10d | 3d | 5d | 10d | 3d | 5d | 10d | 3d | 5d | 10d |
| **Cell viability on IMR-32 cells** | Percentage (%) | 96.83 | 95.50 | 98.36 | 96.63 | 94 | 98.1 | 92.16 | 93.35 | 95.9 | 1.82 | 0.96 | 0.85 | 2.17 | 0.71 | 1.13 | 2.54 | 0.40 | 3.99 |
| **Cell viability on U87 cells** | Percentage (%) | 96.33 | 100 | 95.97 | 93.6 | 93.6 | 91.8 | 84.9 | 85.1 | 72.53 | 1,77 | 1.94 | 1.43 | 1,95 | 3.96 | 1.87 | 1.99 | 1.85 | 7.92 |
| **General Metabolism on IMR-32 cells** | % of control | 103.33 | 103.73 | 101.33 | 112.23 | 113.05 | 96.61 | 116.26 | 110.41 | 97.57 | 1.91 | 6.98 | 0.86 | 20.89 | 11.89 | 2.11 | 10.07 | 19.9 | 0.91 |
| **General Metabolism on IMR-32 cells** | % of control | 10.4.54 | 98.53 | 105.69 | 107.48 | 96.19 | 113.67 | 122.96 | 85.98 | 150.82 | 2.09 | 1.96 | 1.6 | 17.27 | 3.83 | 7.12 | 18.49 | 5.85 | 27.54 |
| **ATP luminescence on IMR-32 cells** | % of control | — | — | 93.95 | — | — | 102.26 | — | — | 96.94 | — | — | 5.23 | — | — | 1.92 | — | — | 2.90 |
| **ATP luminescence on U87 cells** | % of control | __ | __ | 99.76 | __ | __ | 77.94 | __ | __ | 75.31 | __ | __ | 0.40 | __ | __ | 2.47 | __ | __ | 1.98 |
| **Caspase 3-7 on U87 glial cells** | % of control | — | — | 105.82 | — | — | 99.10 | — | — | 119.04 | — | — | 5.04 | — | — | 18.83 | — | — | 21.90 |
| **Membrane Integrity on U87 glial cells** | % of control | __ | __ | 99.71 | __ | __ | 109.20 | __ | __ | 149.38 | __ | __ | 3.02 | __ | __ | 6.41 | __ | __ | 25 |
| **Nuclei area on IMR-32 cells** | µm^2^ | — | — | 68.73 | — | — | 81.42 | — | — | 73.91 | — | — | 3.06 | — | — | 2.61 | — | — | 12.35 |
| **Nuclei area on U87 cells** | µm^2^ | __ | __ | 180.26 | __ | __ | 176.19 | __ | __ | 174.62 | __ | __ | 16.43 | __ | __ | 3.68 | __ | __ | 5.92 |
| **Nuclei perimeter on IMR-32 cells** | µm^2^ | — | — | 30.38 | — | — | 34.06 | — | — | 31.97 | — | — | 0.76 | — | — | 0.47 | — | — | 3.43 |
| **Nuclei area on U87 cells** | µm^2^ | __ | __ | 49.12 | __ | __ | 48.28 | __ | __ | 48.34 | __ | __ | 2.04 | __ | __ | 0.49 | __ | __ | 0.25 |
| **Round index on IMR-32 cells** | µm^2^ | __ | __ | 0.78 | __ | __ | 0.80 | __ | __ | 0.78 | __ | __ | 0.02 | __ | __ | 5 | __ | __ | 14 |
| **Round index on U87 cells** | µm^2^ | __ | __ | 0.81 | __ | __ | 0.80 | __ | __ | 0.81 | __ | __ | 8 | __ | __ | 8 | __ | __ | 9 |
| **ROS luminescence on U87 cells** | % of control / viable cells | __ | __ | 1121.65 | __ | __ | 1163.53 | __ | __ | 1209.05 | __ | __ | 249.68 | __ | __ | 303.02 | __ | __ | 371.94 |
| **TBARS on U87 cels** | µM / mg protein | __ | __ | 0.74 | __ | __ | 0.71 | __ | __ | 0.71 | __ | __ | 0.08 | __ | __ | 104 | __ | __ | 0.08 |
| **GSH/GSSG ratio** | µM | __ | __ | 182.19 | __ | __ | 134.48 | __ | __ | 93.54 | __ | __ | 6.16 | __ | __ | 6.66 | __ | __ | 26.02 |
| **DNA damage on U87 cells (Head)** | % of DNA | __ | __ | 85.91 | __ | __ | 72.53 | __ | __ | 63.08 | __ | __ | 4.46 | __ | __ | 5.5 | __ | __ | 6.89 |
| **DNA damage on U87 cells (Tail)** | % of DNA | __ | __ | 14.06 | __ | __ | 20.16 | __ | __ | 29.16 | __ | __ | 4.47 | __ | __ | 6.35 | __ | __ | 6.89 |

S1 Table: Curve time-response on IMR-32 and U87 cells for 3,5 and 10 days of F exposure (Cell viability and General cell metabolism). Further analysis to access fluoride toxicity were performed only after 10 days of F exposure (ATP, Membrane integrity and cascade 3/7 and Morphometric analysis’ (nuclei area, nuclei perimeter and round index)). Oxidative stress parameters were evaluated only on U87 glial cells after 10 days of exposure (ROS, TBARS, GSH/GSSG, DNA damage). Results are expressed as mean and SD: Standard deviation.
